# Editorial: Assessing the consequences of childhood trauma on behavioral issues and mental health outcomes

**DOI:** 10.3389/fpsyt.2022.1101099

**Published:** 2022-12-02

**Authors:** Myriam V. Thoma, Shauna L. Rohner, Jan Höltge

**Affiliations:** ^1^Psychopathology and Clinical Intervention, Institute of Psychology, University of Zürich, Zurich, Switzerland; ^2^University Research Priority Program “Dynamics of Healthy Aging”, University of Zürich, Zurich, Switzerland; ^3^Department of Psychology, University of Hawai'i at Mānoa, Honolulu, HI, United States; ^4^School of Social Work, Resilience Research Centre, Dalhousie University, Halifax, NS, Canada

**Keywords:** childhood trauma, mental health, behavioral issues, psychosocial adjustment, lifespan impact

## Introduction

Childhood trauma is a common and global phenomenon (1). Multiple reports have demonstrated a significant relationship between childhood trauma, detrimental mental and physical health outcomes, behavioral issues, and psychosocial maladjustment (2). Given its widespread prevalence and harmful health consequences, childhood trauma is a major public health concern. This Research Topic aimed to enhance the understanding of the relationship between childhood trauma and various health outcomes across the life span and in different countries around the globe. Thus, the included papers investigated links between childhood trauma and health outcomes, with seven in China (Chen; Goh et al.; Li et al.; Huang, Yuan et al.; Huang, Song et al.; Shi et al.; Xie et al.), four in Germany (Kampling et al.; Petersen et al.; Schulz et al.; Weitkämper et al.), and one each in Brazil (Muniz Magalhães et al.), Italy (Rossi et al.), and Switzerland (Lau et al.). In addition, this Research Topic contains one conceptual framework developed in United States (Cruz et al.).

The following papers addressed the relationships between childhood trauma and various mood and anxiety outcomes. Huang, Song et al. explored how Qi-stagnation constitution (QSC; unbalanced constitution due to long-term emotional dysfunction and stagnation of Qi movement) and emotion regulation affect the relationship between childhood maltreatment and depressive symptoms. Results revealed a mediating role of QSC between childhood maltreatment and depressive symptoms and a moderating effect of emotion dysregulation on the association between QSC and depressive symptoms. Li et al. investigated if the social comparison emotions of benign and malicious envy can explain the relationship between child maltreatment and depression and anxiety. While malicious envy increased the detrimental effect of child maltreatment on depression and anxiety, benign envy attenuated these effects. Muniz Magalhães et al. analyzed if the experience of childhood sexual abuse was linked to a reduced effect of adjunctive subcutaneous esketamine for the treatment of depression in outpatients who showed resistance to conventional antidepressants. The authors found that childhood sexual abuse might not attenuate the effect of esketamine on depression in this sample, providing evidence for an alternative treatment for otherwise treatment-resistant depressive patients. Petersen et al. investigated the potential interaction of adverse childhood experiences (ACEs), depression, and economic burdens. Results show that a higher number of ACEs was linked to more depressive symptoms. Individuals who had experienced four or more ACEs showed the highest number of depressive symptoms. Moreover, depression acted as a moderator by increasing economic burden. Shi et al. assessed underlying pathways of the relationship between childhood trauma and the fear of missing out (FOMO). Their findings show that childhood trauma was positively associated with FOMO. Furthermore, the results indicate three mediating pathways on this relationship through neuroticism and social anxiety, showing a sequential mediation effect of these two constructs.

The next group of papers investigated the relationship between ACEs and outcomes specifically associated with (developmental) trauma. Cruz et al. discuss recent research on developmental trauma (disorder) and implications for research and practice. The authors also shed light on different risks regarding health and wellbeing for those affected by developmental trauma. Developmental trauma may affect attachment, worldview assumptions, self-perception, and emotion regulation, as well various physical and mental health disorders. The authors highlight the importance of the correct diagnostic process, clinical assessment, and treatment methods for those affected by developmental trauma disorder. Kampling et al. examined the mediating role of personality functioning and epistemic trust in the relationship between ACEs and posttraumatic stress disorder (PTSD) and complex (C)PTSD. Personality functioning partially mediated the relationship between ACEs and PTSD/CPTSD. When epistemic trust was included as a predictor for personality functioning, the explained variance of personality functioning increased, compared to the inclusion of ACEs as a single predictor. Rossi et al. assessed the factor and symptom structure of the Italian version of the International Trauma Questionnaire (ITQ), which measures (C)PTSD symptoms. Findings confirm the factorial validity of the ITQ and support a differentiation between symptoms of PTSD and the three CPTSD symptoms.

The following group of papers investigated various relationships between ACEs and schizophrenia spectrum disorder (SSD). Lau et al. examined whether child maltreatment in Swiss offender patients with SDD could explain the extent of offending behavior, psychopathology, and treatment success. Patients with SDD and no child maltreatment experiences showed the highest levels of psychopathology and violent offending in the past and were most likely to show criminal behavior in the future. Weitkämper et al. assessed prevalence rates of common types of childhood maltreatment in individuals with a primary diagnosis of SSD. While they found generally high rates of childhood maltreatment in individuals with SSD, the prevalence rates for physical abuse and neglect and emotional abuse varied depending on the cut-off thresholds applied.

A last group of studies examined the relationship between ACEs and survivors' behavioral problems and harmful behaviors directed toward themselves or others. The study by Chen found strong associations between ACEs and behavioral problems (e.g., hyperactivity, prosocial problems). An investigation of subgroup differences revealed that males, rural children, and children of mothers with lower education levels were more likely to have been exposed to multiple types of ACEs. Goh et al. conducted a group comparison of individuals convicted for homicide with a non-offending group to investigate if the level of plasma oxytocin could explain the link between childhood trauma and overt aggression. While childhood trauma was negatively associated with plasma oxytocin and positively associated with aggression in both groups, plasma oxytocin was only significantly associated with aggression in the offenders group. Huang, Yuan et al. examined the relationship between childhood trauma and the age of first-time drug use. Younger age of first-time drug use among methamphetamine-dependent patients was linked to more severe childhood trauma, particularly physical and emotional abuse. The results further suggest that a detrimental family environment (e.g., high levels of family conflict and independence), was linked to more severe childhood trauma. Xie et al. investigated if the positive link between child maltreatment and suicidal ideation, planning, and attempts varies by sex; and whether these sex differences can be explained by biological rhythm disorder. The authors found that adolescents with higher levels of biological rhythm disorder and child maltreatment report significantly more suicidal behavior. The extent of the biological rhythm disorder mainly played a significant role for suicidal behaviors in females.

Finally, the study by Schulz et al. shed light on underlying socio-political mechanisms of ACEs by assessing ACE frequencies in individuals who grew up in East or West Germany or abroad. Using two representative samples, they found significantly more ACEs reported by those who grew up abroad, with the lowest rates of ACEs reported by those who grew up in East Germany. They also identified significant age cohort and gender effects.

## Conclusion

This Research Topic highlights recent findings on childhood trauma and various health outcomes across the lifespan and in several countries around the globe. The selected papers help to enhance the awareness of the detrimental link between childhood trauma and various mood and anxiety disorders, outcomes specifically associated with (developmental) stress (disorders), schizophrenia spectrum disorders, behavioral problems, as well as harmful behaviors directed toward themselves or others. These papers have also elucidated various potentially modifiable points of intervention to help diminish these detrimental health consequences of childhood trauma. In order to increase the understanding of the link between childhood trauma and detrimental health outcomes, it appears to be necessary to consider various socio-demographic and person-related factors, as well as aspects of the child-rearing environment, including the wider socio-political context. We believe that this Research Topic provides an up-to-date overview of some of the latest findings of the global and common risk factor that is “childhood trauma” and further advances the understanding of its detrimental health consequences.

## Author contributions

All authors listed have made a substantial, direct, and intellectual contribution to the work and approved it for publication.

## Conflict of interest

The authors declare that the research was conducted in the absence of any commercial or financial relationships that could be construed as a potential conflict of interest.

## Publisher's note

All claims expressed in this article are solely those of the authors and do not necessarily represent those of their affiliated organizations, or those of the publisher, the editors and the reviewers. Any product that may be evaluated in this article, or claim that may be made by its manufacturer, is not guaranteed or endorsed by the publisher.
